# Alkyl Caffeates Improve the Antioxidant Activity, Antitumor Property and Oxidation Stability of Edible Oil

**DOI:** 10.1371/journal.pone.0095909

**Published:** 2014-04-23

**Authors:** Jun Wang, Shuang-Shuang Gu, Na Pang, Fang-Qin Wang, Fei Pang, Hong-Sheng Cui, Xiang-Yang Wu, Fu-An Wu

**Affiliations:** 1 School of Biotechnology, Jiangsu University of Science and Technology, Zhenjiang, P R China; 2 School of the Environment, Jiangsu University, Zhenjiang, P R China; 3 Sericultural Research Institute, Chinese Academy of Agricultural Sciences, Zhenjiang, P R China; Louisiana State University Health Sciences Center, United States of America

## Abstract

Caffeic acid (CA) is distributed widely in nature and possesses strong antioxidant activity. However, CA has lower solubility in non-polar media, which limits its application in fat-soluble food. To increase the lipophilicity of natural antioxidant CA, a series of alkyl caffeates were synthesized and their antioxidant and antitumor activities were investigated. The antioxidant parameters, including the induction period, acid value and unsaturated fatty acid content, of the alkyl caffeates in edible oil were firstly investigated. The results indicated that alkyl caffeates had a lower DPPH IC_50_ (14–23* µ*M) compared to CA, dibutyl hydroxy toluene (BHT) and Vitamin C (24–51* µ*M), and significantly inhibited four human cancer cells (SW620, SW480, SGC7901 and HepG2) with inhibition ratio of 71.4–78.0% by a MTT assay. With regard to the induction period and acid value assays, methyl and butyl caffeates had higher abilities than BHT to restrain the oxidation process and improve the stability of edible oil. The addition of ethyl caffeate to oil allowed maintenance of a higher unsaturated fatty acid methyl ester content (68.53%) at high temperatures. Overall, the alkyl caffeats with short chain length (n<5) assessed better oxidative stability than those with long chain length. To date, this is the first report to the correlations among the antioxidant activity, anticancer activity and oxidative stability of alkyl caffeates.

## Introduction

Currently, synthetic liposoluble antioxidants are still widely used in the edible oil industry because of their efficient antioxidizability. However, various studies have reported that some synthetic fat-soluble antioxidants, such as dibutyl hydroxy toluene (BHT) and butylated hydroxyanisole (BHA), have deleterious effects on human liver, spleen and lung activities as well as the capability to induce carcinoma [1]. Thus, an increasing number of researchers screen healthy and green antioxidants from natural products to investigate their application instead of synthetic antioxidants in oily food [2], [3]. In plants, fruits and vegetables, numerous hydroxycinnamic acids are found, including caffeic acid (CA) [4], *p*-coumaric acid [5] and ferulic acid [6]. Among these hydroxycinnamic acids, increasing interest has been focused on CA because of its broader biological activities, including antioxidative [2], antimicrobial [7] and antitumor [8] activities. CA attracts more interest than synthetic antioxidants due to less damage caused to the human body [9]. However, as an efficient antioxidant, the lower solubility of CA in non-polar media limits its application in fat-soluble food, especially in various edible oils [10], [11]. Kumar and Kanwar [12] reported that hydroxycinnamic esters have stronger lipid solubility in non-polar solvents than hydroxycinnamic acid. Thus, alkyl caffeates are more likely to be applied in oily food as antioxidants [13] because alkyl caffeates possess even higher antioxidative activity as antioxidants [14].

In addition to lipid solubility and higher antioxidizability, a series of studies have been performed to investigate the potential bioactivities of alkyl caffeates. Several researchers have reported the antinociceptive activity [11] and inhibitory effect of alkyl caffeates on lipopolysaccharide-induced nitric oxide production [15]. Ca'rdenas et al [16] studied the antitumor activities of butyl caffeate and ethyl caffeate in different human and murine cell lines. Chou et al [17] also investigated the anticancer activity of caffeate derivatives in human cancer and found that caffeate derivatives decrease the population growth of Colo205 cells. Although the inhibitory effects of caffeate derivatives on cancer cells have been partly investigated, the number of alkyl caffeates and cancer cell types used in the previous experiments was low. Thus, these previous results do not completely reflect the inhibition of different alkyl caffeates on various human cancer cells. In this paper, additional studies were performed to explore the antitumor activities of ten alkyl caffeates with different chain lengths in different human cellular lines.

The oxidation stability of edible oil has been a recent concern, especially its storage life, which is important to maintain oil quality. A variety of factors have important influences on the effectiveness of antioxidants, including antioxidant type, antioxidant amount, storage conditions and edible oil fatty acid profile [18]. It is clear that the typical oxidation chain reaction in edible oil consists of three steps, namely initiation, propagation and termination [19]. In the initiation step, the removal of hydrogen from oil forms a carbon free radical (R-). When oxygen is present, a peroxy radical (ROO·) and hydroperoxide (ROOH) are formed in the propagation step. The hydroperoxide (ROOH) continues the propagation cycle until two free radicals react with each other to produce stable products, such as shorter chain carboxylic acids, sediments, aldehydes and insoluble lipids, in the termination step [20]. These products degrade the quality of food and shorten the storage life. Moreover, the health of the human body is threatened by these stable products. Therefore, higher antioxidative ability is essential to maintain food safety, and antioxidants are necessary to inhibit the oxidation reaction, which implies that more attention should be focused on the utility function of alkyl caffeates as liposoluble antioxidants.

In this paper, the antioxidizability and antiproliferative activities of alkyl caffeates as antioxidants were tested using DPPH and MTT assays, respectively. The practical application of alkyl caffeates as liposoluble antioxidants in edible oil (canola oil, peanut oil, and silkworm pupae oil) was first developed, and several parameters, including the induction period, acid value and effect on unsaturated fatty acids, were systematically investigated.

## Materials and Methods

### Materials and chemicals

**Figure 1:**
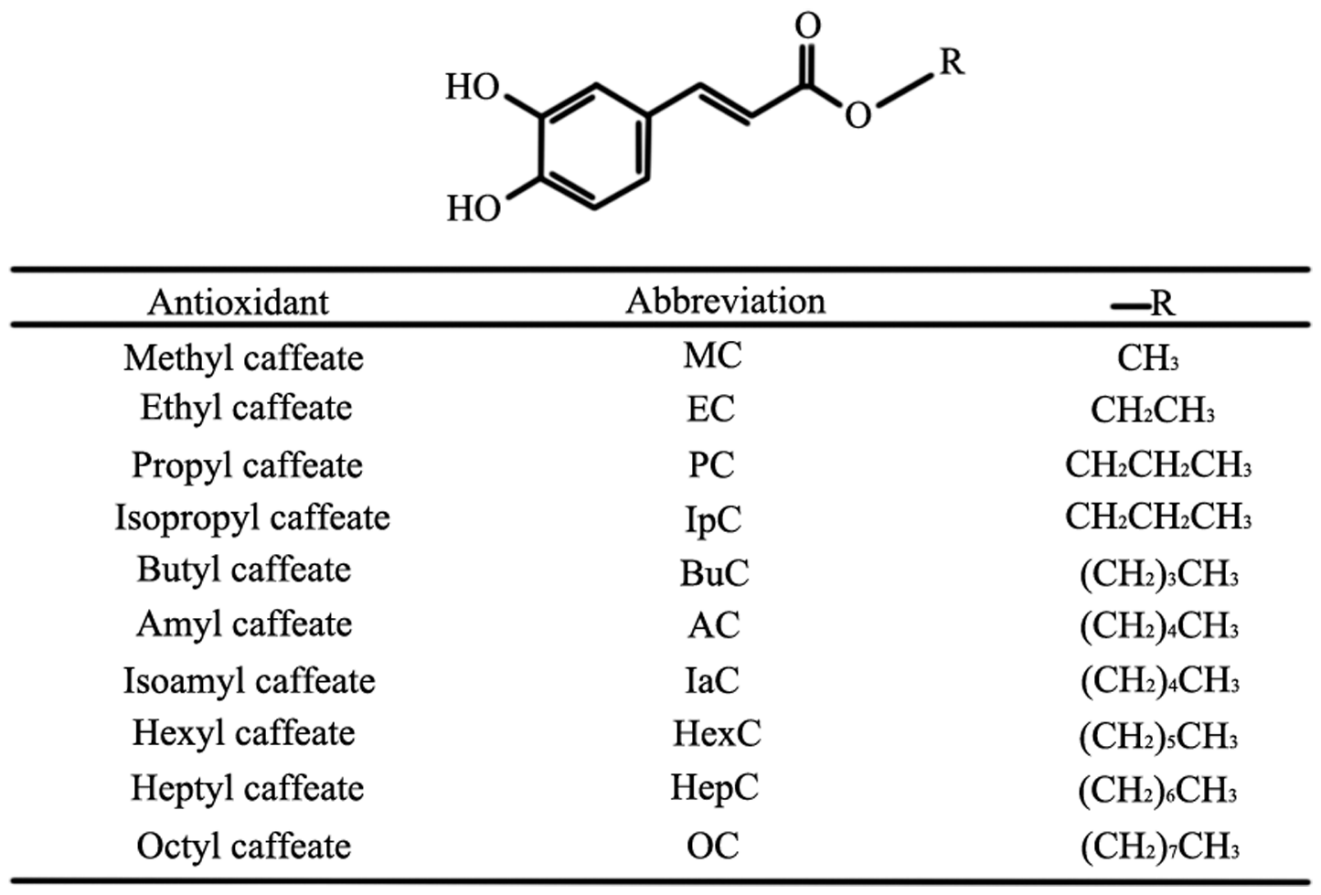
The chemical structures of the alkyl caffeates.

BHT was acquired from Sinopharm Chemical Reagent Co. Ltd. (Shanghai, China). CA was purchased from Nanjing Zelang Pharmaceutical Sci. & Tech. Co. Ltd. (Nanjing, China). 1,1-diphenyl-2-picrylhydrazyl radical (DPPH), 3-[4,5-dimethyl-thiazol-2-yl]-2,5-diphenyltetrazolium bromide (MTT) and caffeic acid phenethyl ester (CAPE) were purchased from Sigma–Aldrich (Gillingham, UK). Alkyl caffeates, including methyl caffeate (MC, purity 99%), ethyl caffeate (EC, purity 99%), propyl caffeate (PC, purity 98%), isopropyl caffeate (IpC, purity 98%), butyl caffeate (BuC, purity 97%), amyl caffeate (AC, purity 97%), isoamyl caffeate (IaC, purity 97%), hexyl caffeate (HexC, purity 98%), heptyl caffeate (HepC, purity 97%) and octyl caffeate (OC, purity 98%), were synthesized via a chemical method using *p*-toluenesulfonic acid as a catalyst [21], [22]. A specific description of the synthesis, purification method, and % yield of alkyl caffeats was placed in File S1, and their HPLC chromatograms were listed as the Figures S1 - S10. Fig. 1 shows the structures of the alkyl caffeates, which have the same mother nucleus. The following alkyl caffeate structures were verified using ^1^H-NMR, which were in accordance with reference [15], [23].

In this investigation, the edible oils included canola oil, peanut oil, and silkworm pupae oil, and these oils were self-produced without additives. The number of antioxidants added to the edible oil was according to the national standards to prolong the storage life (GB 2760–2011, China).

### DPPH assay

The DPPH-scavenging assay was performed to elucidate the radical-scavenging property of CA and its alkyl derivatives. The ethanol solution of DPPH is purple and has an absorption maximum at 517 nm. Briefly, the alkyl caffeates were dissolved using dimethyl sulfoxide (DMSO), and different concentrations were mixed with a methanolic solution of DPPH. The mixtures were then incubated for 30 min in the dark at room temperature [15]. In the presence of antioxidants, DPPH captures an electron and pairs with the free electron, and the color is lost when this electron in balanced with the absorption maximum at 517 nm [24].

Evaluation of the antitumor activity of alkyl caffeates

### Cell culture

The colon cancer (SW620 and SW480), gastric cancer (SGC7901) and hepatic carcinoma (HepG2) cell lines were a generous gift from Jiangsu University (Zhenjiang, China). The original commercial source was from Shanghai Cancer Institute (Shanghai, China). Cells were maintained in Dulbecco's Modified Eagle Medium (DMEM) supplemented with 5% fetal bovine serum and were cultured in flasks at 37 °C in humidified air containing 5% CO_2_. Two-thirds of the growth medium was changed every 2–3 days.

### MTT assay

The MTT assay was performed as previously described [25]. The detection principle of the MTT assay is as follows: succinate dehydrogenase in the mitochondria of living cells enables exogenous MTT to reduce the water insoluble violet crystal, formazan, resulting in formazan deposits in the cells, but dead cells do not have this function, resulting in a lack of cellular formazan deposits. Cancer cells were plated in 96-well microtiter plates at a density of 1×10^4^ cells/well. After 24 h, the culture medium was replaced with 20* µ*L serial dilutions (10–400* µ*M) of alkyl caffeates, and the cells were incubated for 48 h. Sterile filtered MTT solution (100* µ*L; 5 mg/mL) in phosphate buffered saline was added to each well, reaching a final concentration of 0.5 mg MTT/mL. After 4 h, the unreacted dye was removed, and the insoluble formazan crystals were dissolved in DMSO (200* µ*L/well). The plate was measured at 570 nm using a Thermo Electron Corporation Spectrometer [17].

### Rancimat method

The Rancimat method was used to evaluate the induction period (IP) of different edible oils (the canola oil, peanut oil and silkworm pupae oil). In addition to the induction period, the oxidative stability of various oils with and without antioxidants was evaluated by the acid value and unsaturated fatty acid content after high heat. The oxidant stability of canola oil, peanut oil and silkworm pupae oil with and without antioxidants was investigated by a Rancimat (Metrohm 743), and duplicate samples (5.0 g) were analyzed at a constant temperature of 121.6 °C with a stream of purified air at a flow rate of 20 L/h. The induction period of the edible oils corresponded to the inflection point of the electrical conductivity curve.

### Acid value assay

A titration method was used to investigate the acid value of peanut oil and silkworm pupae oil with and without antioxidants. For each sample, the same content of different antioxidants was added to peanut oil or silkworm pupae oil. Approximately 8.0 g of sample was headed using a rotary evaporator with an oil bath at 150 and 180 °C. A sample (3 g) was removed every 20 min with a total of three samples. These samples were dissolved using a mixture of diethyl ether and ethanol, and they were titrated using a phenolphthalein indicator. The titration endpoint was determined using a KOH standard solution (0.05 mol/L).

For each sample, the acid value (AV) was measured based on the titration endpoint and was calculated in mg of KOH per g of sample with the following formula (Eq. 1):
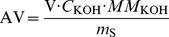
(1)where V is the wasting volume of KOH standard solution when the samples reach the titration endpoint (in mL), *C*_KOH_ is the concentration of the KOH standard solution (in mol/L), *MM*_KOH_ is the molar mass of KOH (in g/mol), and *m*_s_ is the mass of edible oil samples (in g).

Unsaturated fatty acid content assay

Silkworm pupae oil was heated in an oil bath at 180 °C for 7 h. A sample (0.2 g) was dissolved with 0.5 mL of n-hexane in a 10 mL tube. A 2 mol/L KOH-CH_3_OH solution (0.2 mL) was then added to the tube. The solution was then subjected to a whirlpool shock for 1 min, and the sample solution was left for 10 min until the reaction solution was clearly layered. Distilled water (10 mL) was then added, and 1* µ*L of the upper organic solution was collected to dehydrate using anhydrous sodium sulfate. The unsaturated fatty acid content, including linoleate, oleate and linolnate, was measured using gas chromatography (GC) (Agilent 6820). Each methyl ester sample (1* µ*L) was injected at a split ratio of 50∶1. The inlet temperature was 250 °C in the injection port. The GC was equipped with a capillary column (20* µ*m×30 m), and the detector temperature was set at a constant temperature of 280 °C. The oven temperature was programmed to be maintained at 80 °C for 1 min, increased to 220 °C at 5 °C/min, and increased to 250 °C at 2 °C/min. Nitrogen gas was used as the carrier gas at a constant air pressure of 50 kPa.

### Statistical analysis

All experiments were performed in triplicate. The standard deviation of the measures was calculated to check the reliability of the results. Statistical analysis was performed using ANOVA. Statistical comparison between the data was based on the Pearson correlation coefficient, and values less than 0.05 were considered significant. Statistical analysis was performed using SPSS software. Significant differences (*p*<0.05) between the means were determined using Duncan's multiple range tests and multiple comparison tests (LSD).

## Results and Discussion

### Antioxidant activity of alkyl caffeates

**Figure 2:**
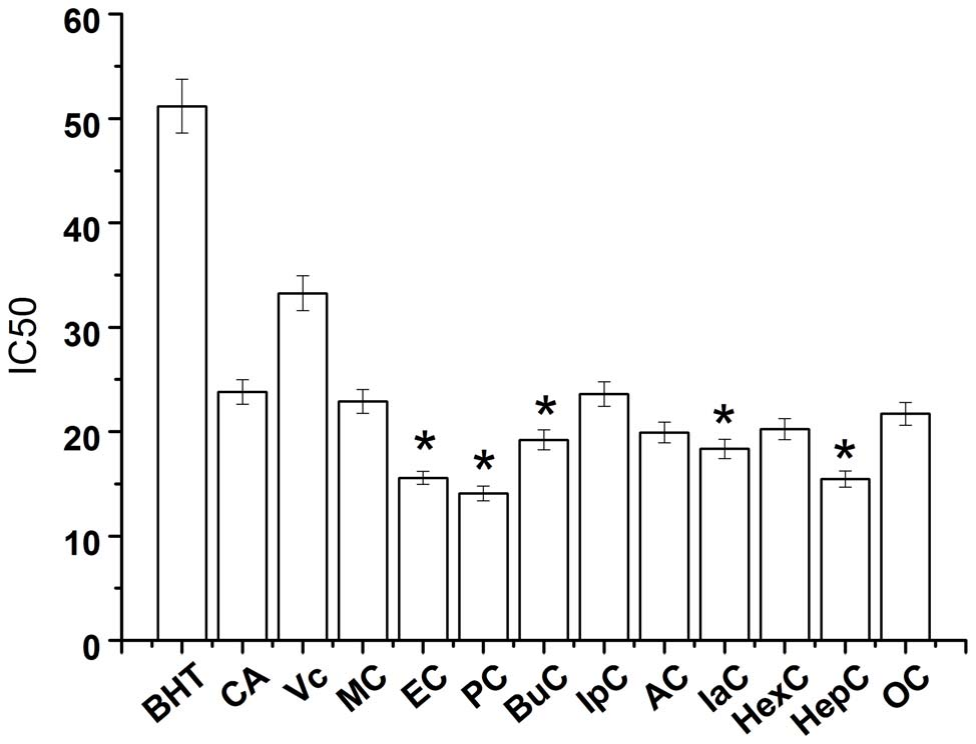
DPPH radical-scavenging activity of different antioxidants. Data are shown as IC_50_ (l* µ*M). The hydroxyl radical-scavenging properties of different antioxidants were determined by spectrophotometry at 517 nm. Results are the mean of three separate determinations ± SEM (error bars), *p*<0.05 vs CA.

Fig. 2 shows the half-inhibition concentration (IC_50_) of the radical-scavenging activity levels with CA and alkyl caffeate with different chain lengths. The traditional antioxidants, namely BHT and Vitamin C (VC), were used for comparison. The inhibition ratio of alkyl caffeates for DPPH radical-scavenging was as follows: PC>EC>HepC>IaC>BuC>AC>HexC>HelC>MC>IpC>CA>VC>BHT. This result indicated that the alkyl caffeates were potent antioxidants because they had higher DPPH scavenging compared to BHT and VC, which are known commercial antioxidants. The IC_50_ values of PC and EC were 14.1 and 15.6* µ*M, respectively, and the IC_50_ values of BHT and VC were 51.2 and 33.3* µ*M, respectively. The antioxidation of PC and EC was approximately 2- and 3-fold more efficient than VC and BHT. In addition, EC, PC, BuC, IaC and HepC showed more significant antioxidation than the parent compound (CA) (*p*<0.05). Other alkyl caffeates, although their effects were not significantly different than CA (*p*>0.05), showed relatively strong antioxidation comparing to CA. Therefore, the alkyl caffeates esterificated from CA and the alkyl alcohols had positive effects on increasing the antioxidizability of CA, which was in accordance with earlier studies [24], [26].

Antitumor activity of alkyl caffeates

**Figure 3:**
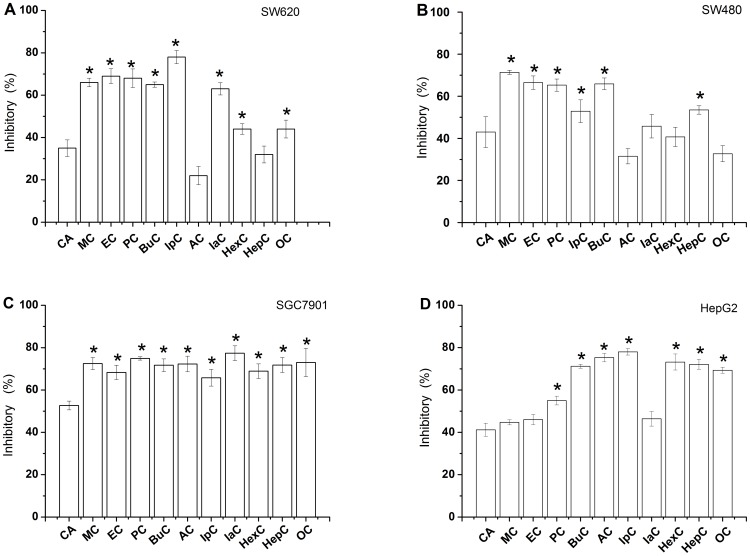
Inhibition ratio of alkyl caffeates and caffeic acid for cancer cells. The cancer cells SW620 (A), SW480 (B), SGC7901 (C) and HepG2 (D) were treated with 200* µ*M alkyl caffeates for 48 h. The reported values are the means of three replicates ± standard deviation, *p<0.05 shows statistical significance when compared to the control group (CA).

Fig. 3A shows the inhibition ratio of alkyl caffeates for SW620 colon cancer cells, and CA was used as the controls. Overall, the alkyl caffeates had higher inhibition ratios compared to CA. In addition, the inhibition ratios of IpC, EC and PC were 78.0±3.1, 69.0±3.5, and 68.0±4.4%, respectively. The majority of the alkyl caffeates, except for AC and HepC, in the growth medium significantly decreased the population of human SW620 colon cells.

Fig. 3B shows the inhibition ratios of the alkyl caffeates for SW480 colon cancer cells, and CA was used for comparison. Most of the alkyl caffeates had higher inhibition ratios compared to CA and CAPE. Furthermore, the inhibition ratios of MC, EC, and BuC were 71.4±1.0, 66.5±3.2, and 65.9±2.8%, respectively. In addition, the result indicated that some alkyl caffeates (MC, EC, PC, IpC, BuC, and HepC) significantly decreased the number of SW480 colon cancer cells.

Fig. 3C shows the inhibition ratios of the alkyl caffeates for SGC7901 gastric cancer cells, and CA was used as the control. The number of gastric cancer cells decreased more significantly after adding alkyl caffeates than after adding CA. These data suggested that the alkyl caffeates had a good inhibition ratio effect on SGC7901 gastric cancer cells. The inhibition rate of IaC was up to 77.4±3.5%. In addition, the inhibition ratios of PC, OC, and MC for the gastric cancer cells were 74.9±0.8, 73.0±6.7, and 72.5±2.9%, respectively. Therefore, it can be concluded that most of the alkyl caffeates had good inhibition ratio effects on SGC7901 gastric cancer cells, which agreed with a previously reported study [16].

Fig. 3D shows the inhibition ratio of alkyl caffeates for HepG2 hepatic carcinoma cells, and CA was used as the control. Overall, the alkyl caffeates had higher inhibition ratios compared to CA. After alkyl caffeates were added to hepatic carcinoma cells, the number of hepatic carcinoma cells robustly decreased. In addition, the inhibition ratios of IpC, AC and HexC were 78.0±1.5, 75.3±2, and 73.2±3.8%, respectively. This result also indicated that alkyl caffeates (PC, IpC, BuC, AC, HexC, HepC, and OC) significantly decreased the number of HepG2 hepatic carcinoma cells.

Thus, all of the alkyl caffeates were cytotoxic to all four cancer cell types. A comparative analysis between SW620 and SW480 cells showed that both cell lines behaved similarly. IaC was more efficient than AC in the SW620 and SW480. This indicated that cell subpopulations had similarly biological properties. However, for the SGC7901 and HepG2 cell, the alkyl caffeates had different behavior. It suggests a selective action against the tumor cells. The cytotoxic effects of alkyl caffeates are concerned with their lipophilicity and the size of the alcoholic moieties. Thus, although IaC and AC have similar molecules, they have different inhibiting effects on different cell lines.

Isopropyl caffeate (IpC) had the highest inhibition ratio (78.0%) for SW620 and HepG2 cancer cells. The SGC7901 and SW480 cells were inhibited by isoamyl caffeate (IaC) and methyl caffeate (MC) with inhibition ratios of 77.4 and 71.4%, respectively. In addition, the alkyl caffeates showed a more pronounced anticancer effect on SGC7901 cells. Thus, it was necessary to further evaluate the concentration-dependent relationship between alkyl caffeates and SGC7901 cells. A cytotoxic effect of alkyl caffeates at different concentrations on SGC7901 cells was observed. Table 1 shows that the inhibition ratio increased with increasing concentrations of alkyl caffeates within a certain concentration range, but any further increase caused the inhibition ratio to decrease. The highest inhibition ratio was obtained with the concentration of 200* µ*M, except for IpC. The inhibition ratios of IaC (77.4%) and PC (74.9%) for SGC-7901 cells were the most significant. In the concentration range of 10 to 200* µ*M, inhibition of the cancer cells gradually decreased with dilutions. Based on these results, the molecular mechanisms involved in the generation of the antitumor action of the caffeate derivatives were further explored. The addition of an alkyl chain on CA molecule might have changed its lipophilicity, which gives new characteristics to the esters, such as the increase of permeability through the cell membrane, a property which could in turn influence the bioavailability [27].

**Table 1:** Inhibition rate (%) of alkyl caffeates for gastric cancer cells at different concentrations.

Compound	Inhibition rate (%)				
	400* µ*M	200* µ*M	100* µ*M	50* µ*M	10* µ*M
MC	69.8±1.8^m^	72.5±2.9	56.7±3.0	34.3±3.6	18.7±3.2
EC	64.6±1.2	68.3±1.9	52.0±2.8	37.3±2.5	28.9±2.7
PC	54.6±1.3	74.9±0.8	55.5±3.2	30.4±2.8	20.3±1.1
IpC	66.8±1.1	65.8±1.3	41.7±1.8	33.5±0.9	12.4±0.8
BuC	64.5±1.9	71.7±2.0	60.0±1.4	55.0±1.6	24.2±3.4
AC	63.5±0.7	72.3±2.3	73.3±1.7	29.7±1.9	8.7±3.9
IaC	68.2±0.8	77.4±3.5	72.4±2.7	35.2±2.7	12.0±2.1
HexC	40.5±1.2	68.9±1.2	64.9±3.3	38.9±3.8	31.3±3.8
HepC	19.2±1.1	71.4±2.1	67.2±0.9	71.8±2.9	58.3±0.7
OC	2.3±1.0	73.0±0.8	70.8±1.3	53.0±0.6	21.9±2.5
CA	45.1±0.9	52.7±1.3	32.2±2.4	15.4±2.6	—

m Each data represents the mean of three replicates ± standard deviation.

### The effect of alkyl caffeates on induction period of edible oil

Among edible oils, canola oil is widely used in the world, and the amount of canola oil used is large. Peanut oil is the characteristic oil in Asian countries, and silkworm pupae oil is a newly developed insect oil. Silkworm pupae oil contains a large amount of unsaturated fatty acids, such as *α*-linolenic acid (ALA) and docosahexaenoic acid (DHA), which can effectively prevent a variety of diseases. Furthermore, these unsaturated fatty acids are easily oxidized [28]. Thus, in the present study, these three edible oil types were selected to test the application of alkyl caffeates as antioxidants and to demonstrate their practical value.

**Figure 4:**
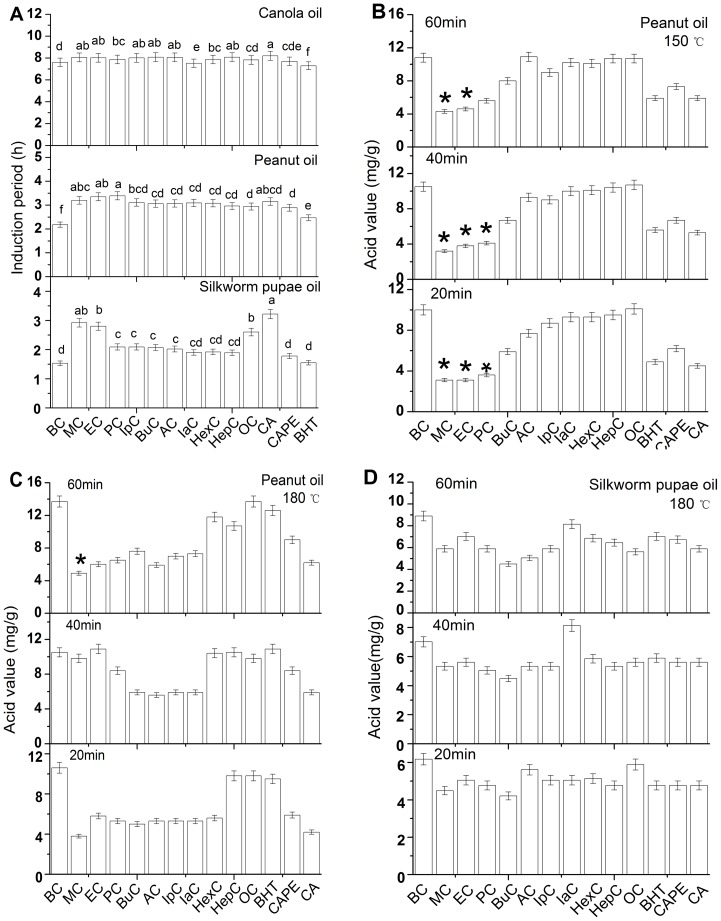
The induction period and acid value of different edible oil with the addition of alkyl caffeates. BC =  blank control. (A) Value of induction period obtained by the Rancimat method for canola oil, peanut oil and silkworm pupae oil with the addition of different antioxidants at 121.6 °C. Different letters indicate significant differences (*p*<0.05) based on BC. (B) Different antioxidants were added to peanut oil, and the acid value was detected after 20 minutes, 40 minutes and 60 minutes of heating at 150 °C. (C) Different antioxidants were added to peanut oil, and the temperature was 180 °C. (D) Acid value of silkworm pupae oil with different antioxidants was detected at the same time intervals at 180 °C. *p<0.05 shows statistical significance when compared to the control group (CA).

Fig. 4A shows the induction periods (IPs) of canola oil, peanut oil and silkworm pupae oil with and without antioxidants. The induction periods of the three edible oils were improved after adding different antioxidants, thereby resulting in prolonged storage life. For silkworm pupae oil, the more effective antioxidants were CA, MC, EC, and OC, and the corresponding induction periods of these antioxidants were twice as long as BHT. For peanut oil, MC, EC, and PC had higher induction periods than the other antioxidants, which indicated that the antioxidizability of these three antioxidants was stronger than the other alkyl caffeates and even BHT, and these antioxidants may prolong the induction period of peanut oil from 1 to 1.5 h. Additionally, the induction period of canola oil with added antioxidants was extended by approximately 0.5 h. As compared to the chemically synthesized antioxidant (BHT), alkyl caffeates and CA were useful to strengthen the oxidation stability of canola oil and to promote perfect antioxidizability. The higher antioxidation of alkyl caffeates and CA may have been due to the presence of the catechol group, which contributes to the stabilization of the phenoxyl radical intermediate [29].

The different letters in Fig. 4A represent significant differences (*p*<0.05) based on the blank control (BC). The results indicated that the induction period differences of all antioxidants, except CAPE and BHT, for canola oil and peanut oil were insignificant. The differences of other antioxidants for the induction periods of silkworm pupae oil were also insignificant.

### The effect of alkyl caffeates on the acid value of edible oil

Figs. 4B and 4C show the acid value (AV) of peanut oil with the addition of alkyl caffeates, CA and BHT or no addition of antioxidants at 150 and 180 °C, respectively. Overall, the acid value of edible oil increased with increasing heating times. However, the acid value of peanut oil with antioxidants was inhibited, and the highest effective antioxidant was MC with heating at 150 °C for 60 min. When the temperature increased to 180 °C, the acid value of peanut oil increased comparatively. It indicated that higher temperatures had greater influence on the storage of edible oil. However, the acid value of peanut oil with some antioxidants, such as MC, EC, and PC, decreased after 40 min suggesting that the storage ability of oil was improved, which agreed with previous reports by Choe E [30] and Tang H [31]. The addition of some antioxidants can improve the oxidation ability of oil over a certain period. Nevertheless, some antioxidants, such as AC, lose their efficiency of antioxidation as the storage time is prolonged [32]. At 180 °C, the peanut oil with added OC had a lower acid value after heating for 60 min. Compared to BHT, the other antioxidants were more effective in improving the storage stability of peanut oil, which may be due to the dissociation energy having an important influence on the transfer of hydrogen to peroxyl radicals [30]. CA and alkyl caffeates formed an *o*-quinone radical, a more stable radical, which generated a conjugated π system through resonance to donate electrons to peroxide radicals [32]. In addition, Fig. 4D shows that the acid value of silkworm pupae oil added some antioxidants was lower than that of peanut oil at 180 °C. Among all the antioxidants, BuC showed the lowest acid value and the highest antioxidation activity after 60 mins of heating at 180 °C.

It was found that when the peanut oil was heated to 150 °C, MC, EC and PC have more significant differences than CA (*p*<0.05). While there was no significant difference for alkyl caffeates in the acid values test of the silkworm pupae oil. Thus, it could be deduced that the esterification did not obviously alter the capacity of CA preventing the oxidation of oils.

### The effect of alkyl caffeates on unsaturated fatty acids in edible oil

Table 2 shows that the addition of antioxidants preserved unsaturated fatty acids, including C18∶1 (oleate), C18∶2 (linoeate), and C18∶3 (linolenate), in silkworm pupae oil. The addition of antioxidants could maintain the content of unsaturated fatty acids at a high temperature for a long time. EC, IpC, IaC, and HepC were more effective in inhibiting the oxidation process compared to BHT. No significant differences (*p*<0.05) in the levels of C16∶0, C18∶0, C18∶1, C18∶2, and C18∶3 were detected among the silkworm pupae oil samples during the 7 h of heating at 180 °C. However, EC in the level of C16∶1 was significant difference comparing with BHT.

**Table 2:** Fatty acid methyl ester composition with the addition of different antioxidants or no addition of antioxidants after 7°C.

Fatty acid composition (%)	Antioxidant												
	BC	MC	EC	PC	IpC	BuC	AC	IaC	HexC	HepC	CA	BHT	CAPE
Palmitic (C16∶0)	^a^28.62±0.01^m^	^a^26.83±0.02	^a^25.40±0.00	^a^27.62±0.02	^a^25.96±0.01	^a^27.09±0.03	^a^28.49±0.04	^a^26.07±0.01	^a^27.21±0.03	^a^26.09±0.01	^a^27.28±0.01	^a^26.65±0.01	^a^26.96±0.02
Palmitoleic (C16∶1)	^b^1.31±0.00	^ab^1.17±0.00	^a^1.15±0.00	^ab^1.20±0.00	^ab^1.23±0.00	^ab^1.16±0.00	^ab^1.24±0.00	^ab^1.23±0.00	^ab^1.18±0.00	^ab^1.23±0.00	^b^1.29±0.00	^ab^1.27±0.00	^ab^1.26±0.00
Stearic (C18∶0)	^a^6.80±0.01	^a^6.37±0.00	^a^6.06±0.00	^a^6.85±0.01	^a^6.12±0.00	^a^6.48±0.01	^a^6.51±0.00	^a^6.24±0.00	^a^6.49±0.01	^a^6.17±0.00	^a^6.67±0.01	^a^6.30±0.00	^a^6.33±0.00
Oleate (C18∶1)	^a^35.43±0.05	^a^36.41±0.01	^a^35.27±0.01	^a^37.00±0.01	^a^35.71±0.01	^a^36.53±0.02	^a^36.74±0.00	^a^36.19±0.00	^a^36.00±0.02	^a^35.95±0.00	^a^37.61±0.03	^a^36.98±0.01	^a^36.44±0.02
Linoeate (C18∶2)	^a^5.15±0.01	^a^5.22±0.00	^a^5.42±0.00	^a^5.16±0.00	^a^5.30±0.00	^a^5.16±0.01	^a^5.04±0.00	^a^5.26±0.00	^a^5.20±0.00	^a^5.26±0.00	^a^5.54±0.01	^a^5.58±0.00	^a^5.16±0.00
Linolenate (C18∶3)	^a^22.68±0.06	^a^23.99±0.03	^a^26.69±0.01	^a^22.18±0.03	^a^25.68±0.02	^a^23.58±0.06	^a^21.97±0.03	^a^25.01±0.01	^a^23.91±0.05	^a^25.30±0.01	^a^21.62±0.06	^a^23.21±0.01	^a^23.85±0.05
Saturated	35.43	33.20	31.47	34.46	32.08	33.57	35.00	32.31	33.70	32.26	33.95	32.95	33.29
Unsaturated	64.57	66.80	68.53	65.54	67.92	66.43	65.00	67.69	66.30	67.74	66.05	67.05	66.71

a–b The mean values in the same line for the silkworm pupae oil under the same heat-up time with different antioxidants are significantly different (*p*<0.05).

m Each data represents the mean of three replicates ± standard deviation.

The highest unsaturated fatty acid methyl ester (FAME) content in silkworm pupae oil was oleate with 35.43±0.05% without the addition of antioxidant. After adding antioxidants, the total unsaturated acid methyl ester content increased. The addition of EC resulted in the highest unsaturated fatty acid methyl ester content of 68.53%. In addition, IpC, HepC, and IaC also improved the antioxidation and maintained a higher unsaturated fatty acid content in silkworm pupae oil with values of 67.92, 67.74, and 67.69%, respectively. EC increased the unsaturated fatty acid content in silkworm pupae oil by 6.13%, and BHT increased the unsaturated fatty acid content by only 3.84%. Thus, EC was able to preserve unsaturated fatty acids in oil after high temperature heating 1.6-fold more than that of BHT, which may be due to the presence of allylic and bis-allylic hydrogens in the ester alkyl chains being beneficial to oxidation stability [33]. Meanwhile, their green source had more advantages as antioxidants in edible oil. Therefore, the addition of alkyl caffeate to edible oil may be a novel potential lipophilic antioxidant and adjuvant against cancer in the future.

### The correlations among the bioactivities of alkyl caffeates

Table 3 shows the correlation among the antioxidant activity, antitumor activity and oxidative stability of alkyl caffeates. Correlations were observed between the antioxidant activity (DPPH assay) and the inhibition of SW480, SGC-7901 and HepG2. The highest positive correlation between the antioxidant activity and antitumor activity appeared in HepG2 cancer cell (R = 0.587). While there were negative correlations between SW480 and SGC-7901, with R value of 0.529 and 0.406, respectively. There was a correlation between the oxidative stability, including the inhibition of acid value of peanut oil at 180 °C and induction periods, and cancer cells. For the inhibition of acid value of peanut oil at 180 °C, R values were −0.81, −0.925 and 0.825 for SW620, SW480 and HepG2, respectively. For the induction periods, R values were 0.492, 0.494 and −0.667 for SW620, SW480 and HepG2, respectively. A correlation was also found between the antioxidant activity and acid values of peanut oil and silkworm pupae oil at 180 °C. The correlation values (R) between the DPPH (the antioxidant activity) and PO180 (the acid value of peanut oil at 180 °C) and SPO180 (the acid value of silkworm pupae oil at 180 °C) were 0.509 and −0.451, respectively.

**Table 3:** The Pearson correlation coefficients among the antioxidant activities, antitumor activities and oxidative stability of alkyl caffeates.

	DPPH a	SW620 b	SW480 c	SGC7901 d	HepG2 e	PO180 f	SPO180 g	SPO IPs h
DPPH	1.000							
SW620	0.032	1.000						
SW480	−0.529	0.829	1.000					
SGC7901	−0.406	0.622	−0.735	1.000				
HepG2	0.587	−0.678	−0.731	−0.642	1.000			
PO180	0.509	−0.810	−0.925	0.274	0.825	1.000		
SPO180	−0.451	0.375	−0.237	0.427	−0.650	0.179	1.000	
SPO IPs	0.292	0.492	0.494	−0.367	−0.667	−0.736	0.290	1.000

a DPPH – the antioxidant activity;

b SW620 – the inhibition of SW620 cancer cell;

c SW480 – the inhibition of SW480 cancer cell;

d SGC7901 – the inhibition of SGC-7901 cancer cell;

e HepG2 – the inhibition of HepG2 cancer cell;

f PO180 – the acid value of peanut oil at 180 °C;

g SPO180 – the acid value of silkworm pupae oil at 180 °C;

h SPO IPs – the induction periods of silkworm pupae oil.

**Figure 5:**
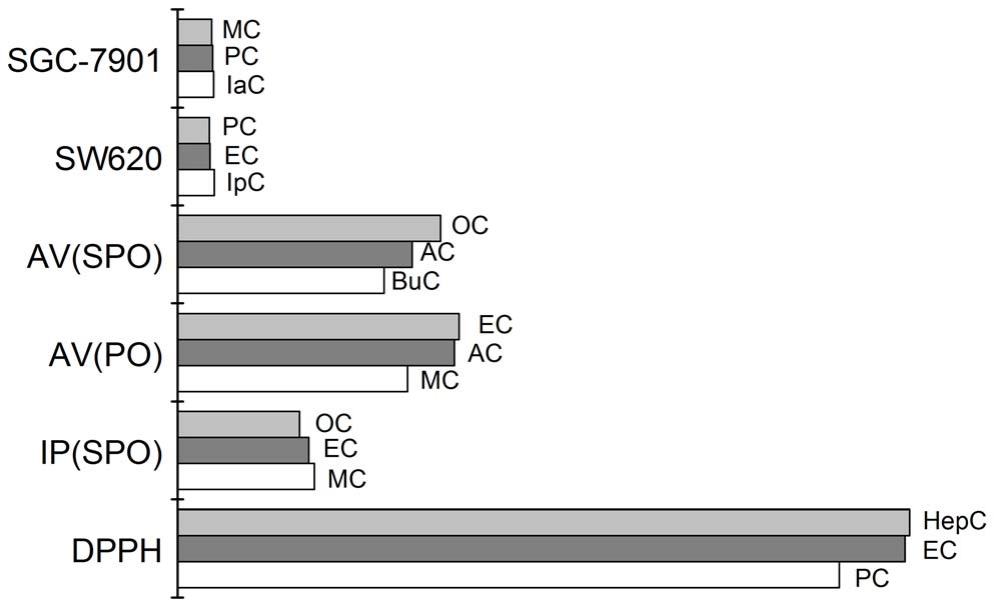
Top 20% of alkyl caffeates for three bioactivities. The bioactivities include antioxidant activity (DPPH), oxidative stability (IP, SPO; AV, PO; AV, SPO) and antitumor activity (HepG2; SW620). DPPH – the antioxidant activity, IP (SPO) – the induction periods of silkworm pupae oil, AV(PO) – the acid value of peanut oil at 180 °C, AV(SPO) – the acid value of silkworm pupae oil at 180 °C, SW620 – the inhibition of SW620 cancer cell, SGC-7901 – the inhibition of SGC-7901 cancer cell.

Fig.5 shows that PC, HepC and EC had better antioxidant activity than other alkyl caffeates through DPPH method. Through the oxidative stability tests for alkyl caffeates in oil, MC, EC and OC had longer induction period than others. Thus, oil added these three alkyl caffeates could prolong shelf-life. For the acid value tests, alkyl caffeates containing shorter chain length, such as MC, AC, PC, assess lower pH value. Similarly, higher unsaturated fatty acid content appeared among MC, PC, IpC and IaC (Table 2). In this sense, alkyl caffeats with short chain length (n<5) assessed better oxidative stability than those with long chain length.

In addition to the correlations of antioxidant activity and oxidative stability about alkyl caffeates, some studies reported that antioxidant and antitumor activities have some connections to some degree [34], [35]. Through our investigation, EC and PC had not only higher antioxidant activity but also antitumor activity. It may be because the chain length will affect antioxidant activity and antitumor activity of alkyl caffeates. Alkyl caffeates with shorter chain length had stronger antioxidant activity and antitumor activity. However, there are some exceptions, such as MC, IpC and IaC. MC had more obvious inhibition ratio for SW480, but its antioxidant activity was not obvious comparing with EC and PC. Similarly, IpC and IaC could apparently inhibit cancer cell proliferation for HepG2 and SGC-7901, respectively. However, their antioxidant activity did not express adequately through DPPH tests. This may be because the cell types, different proliferation and motility in cell population in *vitro* [36] can affect the antitumor activity of alkyl caffeates. For some exception, there may be other explanations that we don't know, so that we are planning to do some investigations *in vivo* to make sure more accurately their mechanism in the correlation of antioxidant and antitumor activities of alkyl caffeates.

Overall,in these alkyl caffeates, MC, EC and PC expressed more obviously influences on these bioactivities not only for antioxidant activity and antitumor activity, but even for improving the oxidative stability of oil. Thus, MC, EC and PC could be as potential lipidic antioxidants and cancer chemopreventive agents in the food and pharmaceutical industries.

## Conclusions

The antioxidant activity, antitumor property and oxidation stability of edible oil with added alkyl caffeates were investigated in the present study. The order of inhibition ratios for DPPH radical scavenging was as follows: PC>EC>HepC>IaC>BuC>AC>HexC>HelC>MC>IpC>CA>VC>BHT. For the antitumor activity, IpC showed the highest inhibition for SW620 and HepG2 cells (inhibition ratios of 78.0±3.1 and 78.0±1.5%, respectively), IaC showed the highest activity for SGC-7901 cells (inhibition ratio of 77.4±3.5%), and MC showed the highest activity for SW480 cells (inhibition ratio of 71.4±1.0%). The edible oil oxidation stability results indicated that all alkyl caffeates more efficiently prolonged the induction period of edible oil compared to BHT with different degrees. Moreover, MC was the most effective antioxidant to decrease the acid value of edible oil at 150 °C, and BuC showed the highest resistance to oxidation at 180 °C. Furthermore, EC effectively maintained the content of unsaturated fatty acid methyl ester with a value of 68.53%, which was 1.6 times greater than that of BHT in silkworm pupae oil after 7 h of heating at 180 °C. Further studies were done to discuss the correlations among the antioxidant activity, anticancer activity and oxidative stability of alkyl caffeates. The highest positive correlation between the antioxidant and antitumor activities appeared in HepG2 cancer cell (R = 0.587). The highest positive correlation between the inhibition of acid value of peanut oil at 180 °C and inhibition of HepG2 cancer cell was 0.825. Therefore, the addition of alkyl caffeate to edible oil may be a novel potential lipophilic antioxidant and adjuvant against cancer.

## Supporting Information

Figure S1 HPLC chromatogram of MC.
(HPLC)

Figure S2 HPLC chromatogram of EC.
(HPLC)

Figure S3 HPLC chromatogram of PC.
(HPLC)

Figure S4 HPLC chromatogram of IpC.
(HPLC)

Figure S5 HPLC chromatogram of BuC.
(HPLC)

Figure S6 HPLC chromatogram of AC.
(HPLC)

Figure S7 HPLC chromatogram of IaC.
(HPLC)

Figure S8 HPLC chromatogram of HexC.
(HPLC)

Figure S9 HPLC chromatogram of HepC.
(HPLC)

Figure S10 HPLC chromatogram of OC.
(HPLC)

File S1 Synthesis and HPLC analysis of alkyl caffeates.
(HPLC)
