# Supplementary figures and images for: Alkyl Caffeates Improve the Antioxidant Activity, Antitumor Property and Oxidation Stability of Edible Oil
Source: PLoS One. 2014 Apr 23;9(4):e95909. doi: 10.1371/journal.pone.0095909 (PMC3997486; doi:10.1371/journal.pone.0095909)

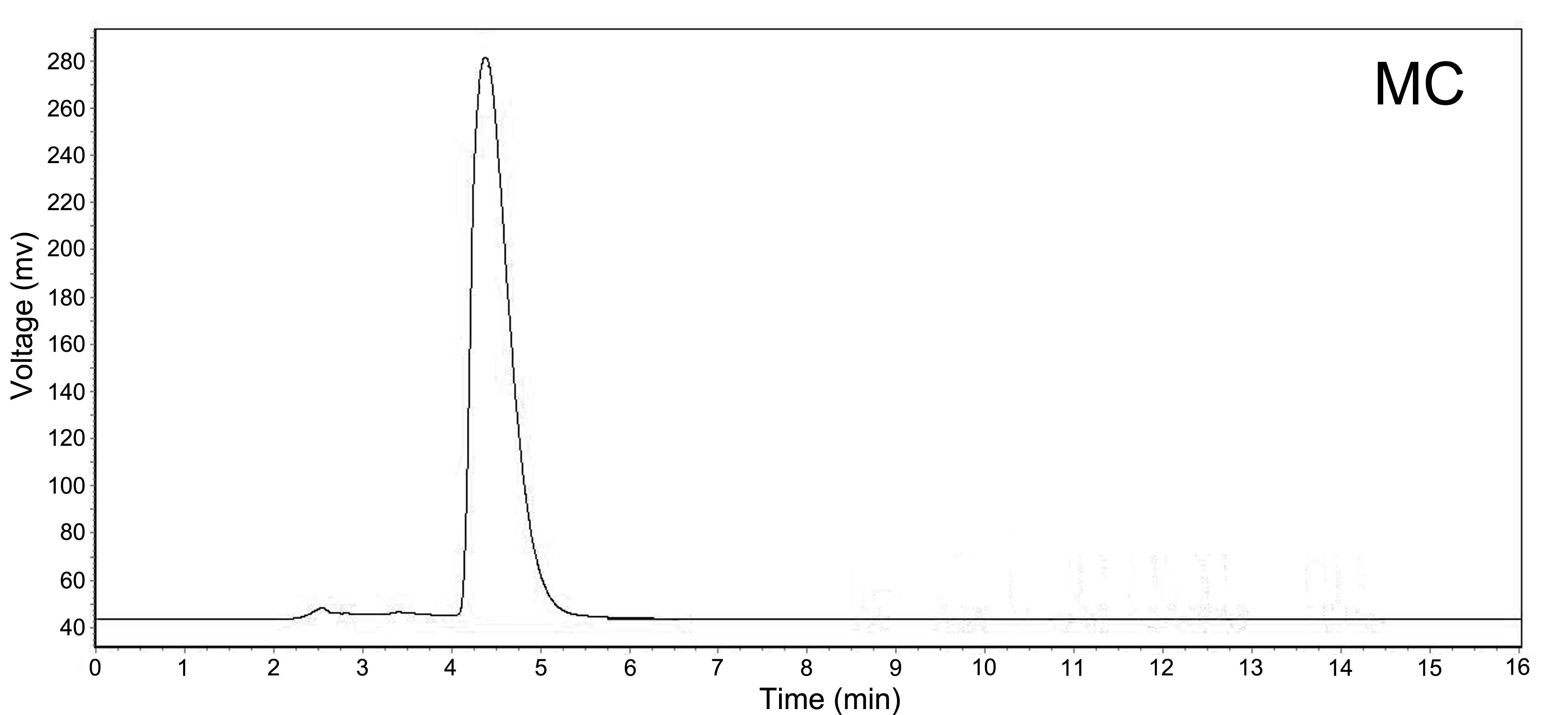

Supplement: Figure S1 — HPLC chromatogram of MC. (HPLC) [file pone.0095909.s001.hplc]

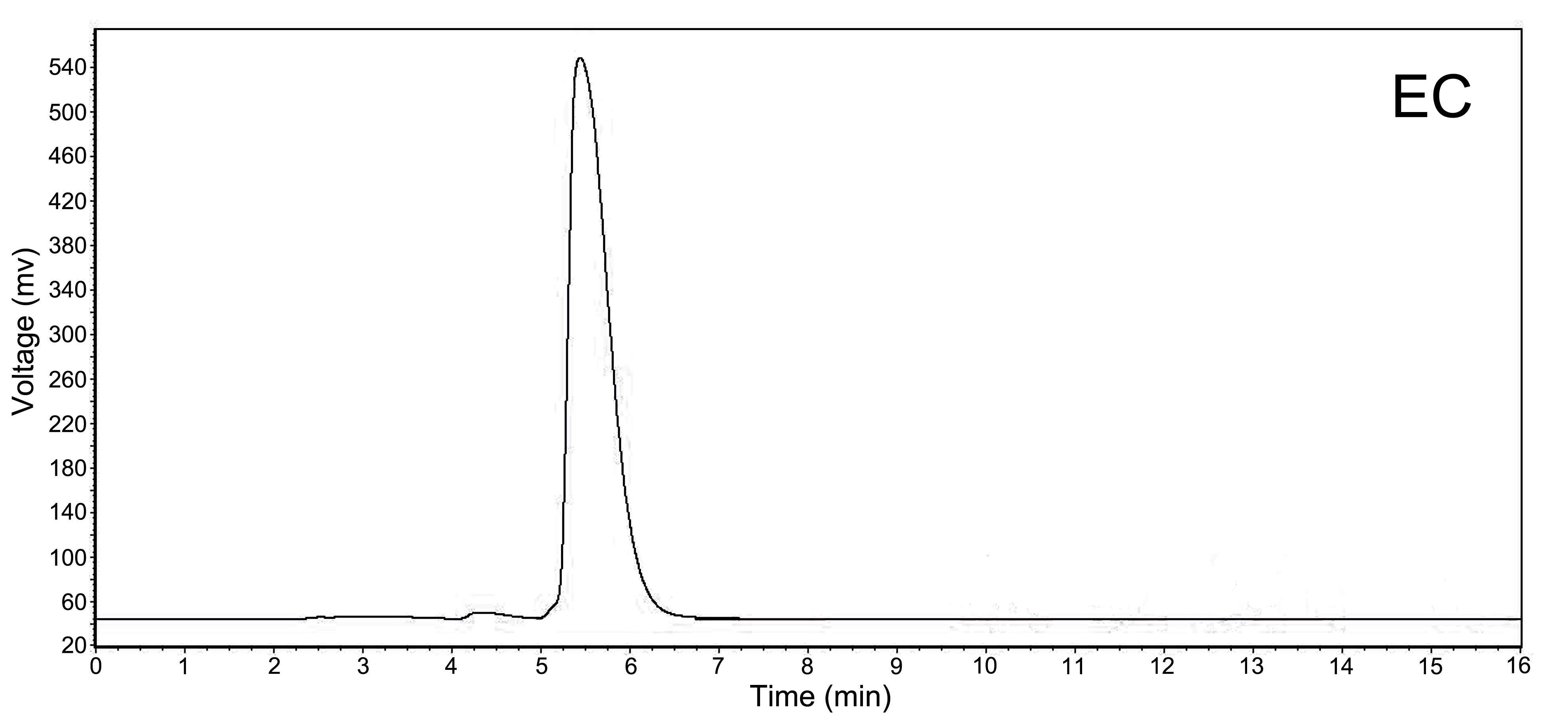

Supplement: Figure S2 — HPLC chromatogram of EC. (HPLC) [file pone.0095909.s002.hplc]

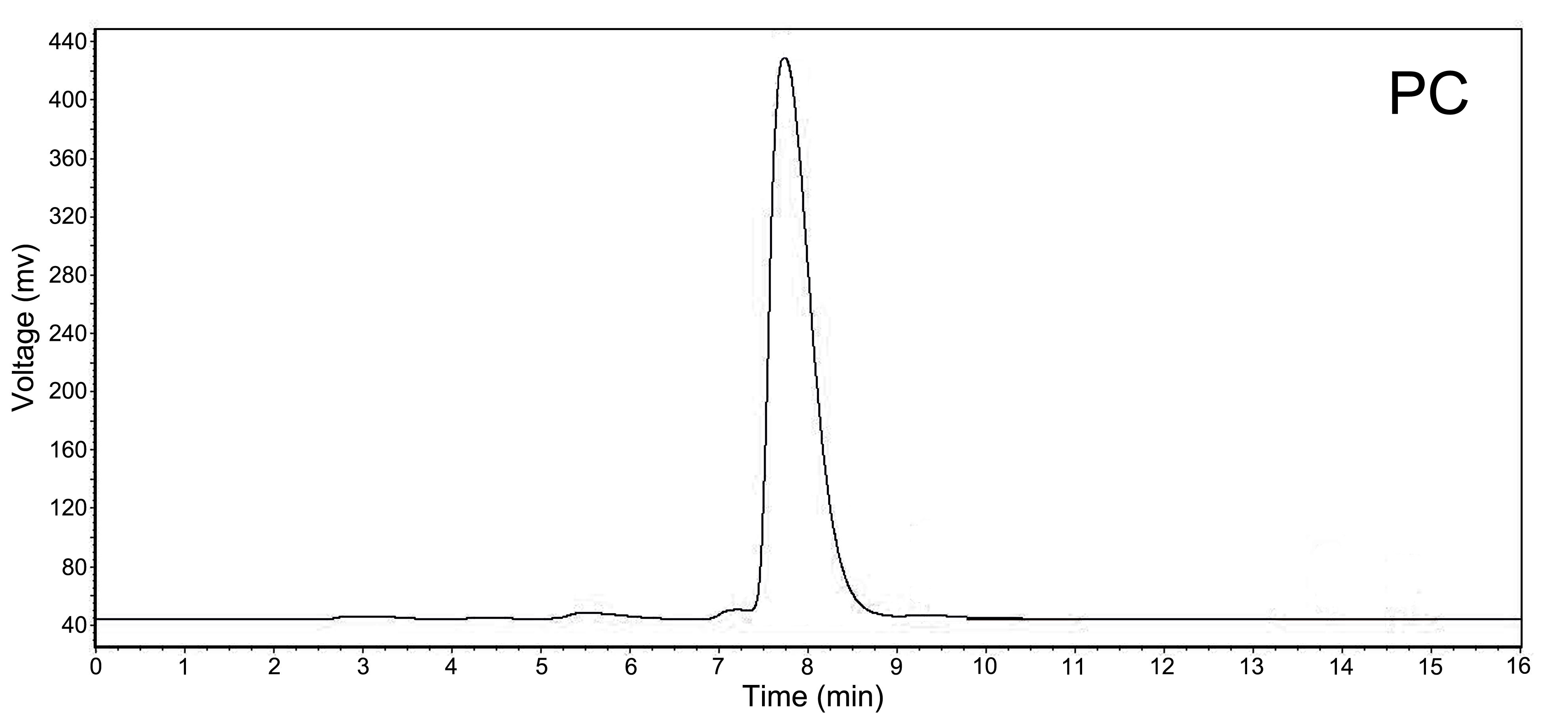

Supplement: Figure S3 — HPLC chromatogram of PC. (HPLC) [file pone.0095909.s003.hplc]

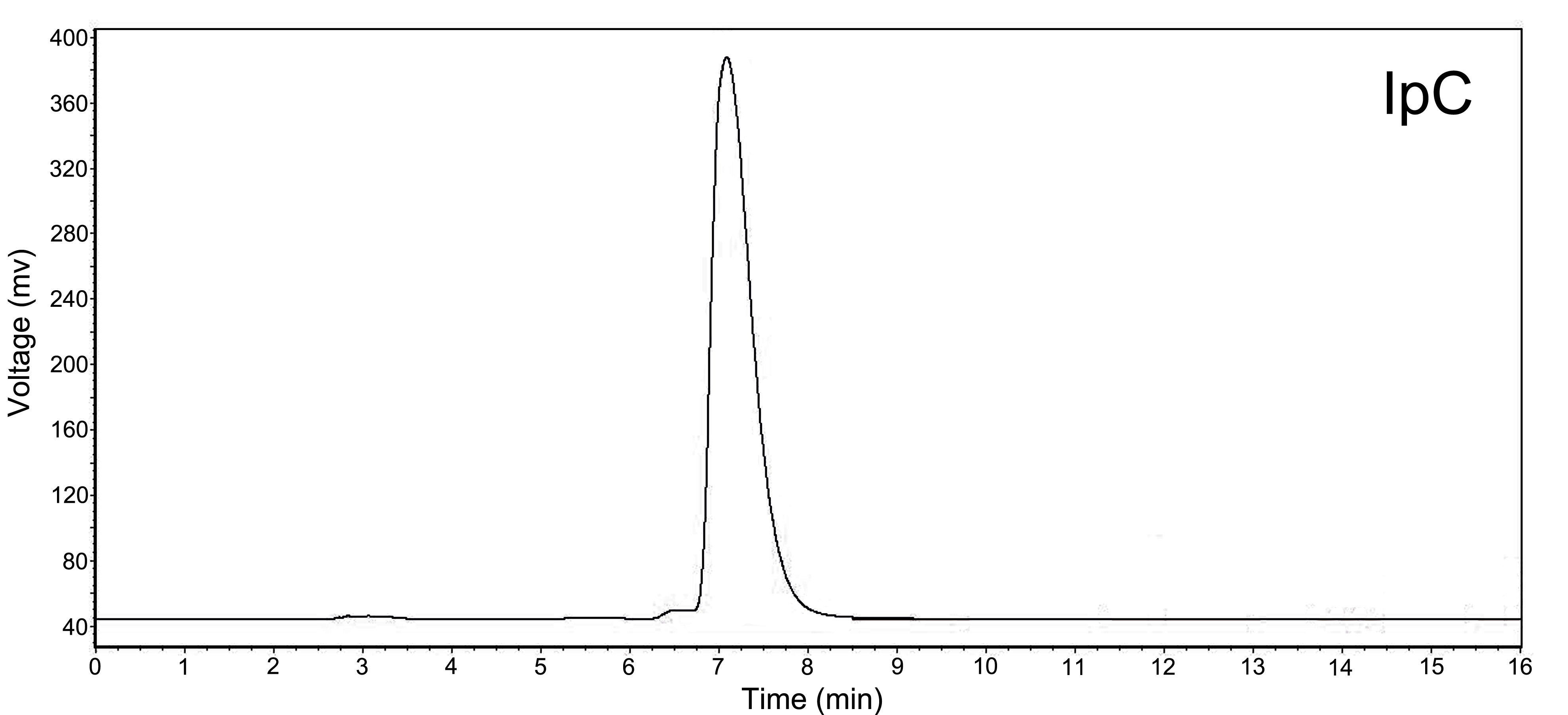

Supplement: Figure S4 — HPLC chromatogram of IpC. (HPLC) [file pone.0095909.s004.hplc]

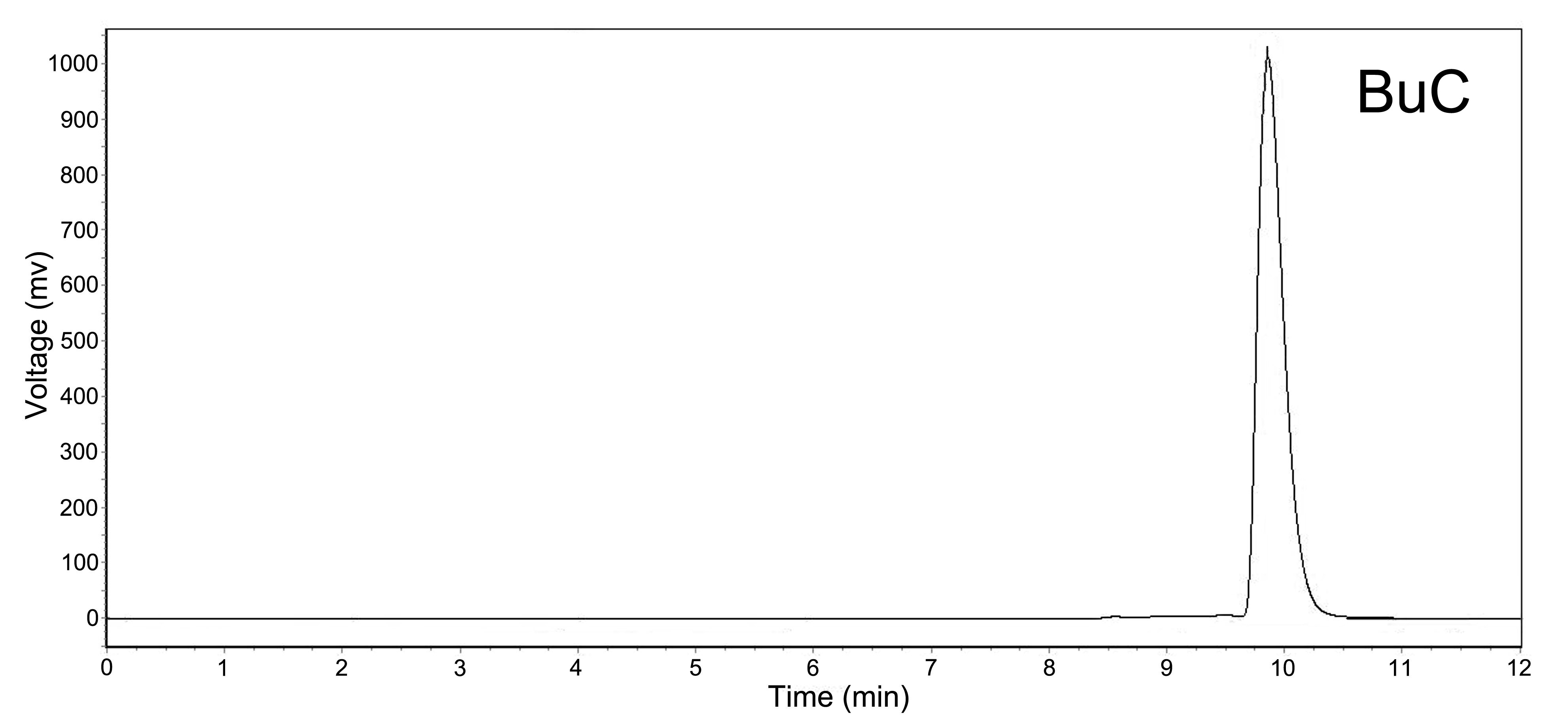

Supplement: Figure S5 — HPLC chromatogram of BuC. (HPLC) [file pone.0095909.s005.hplc]

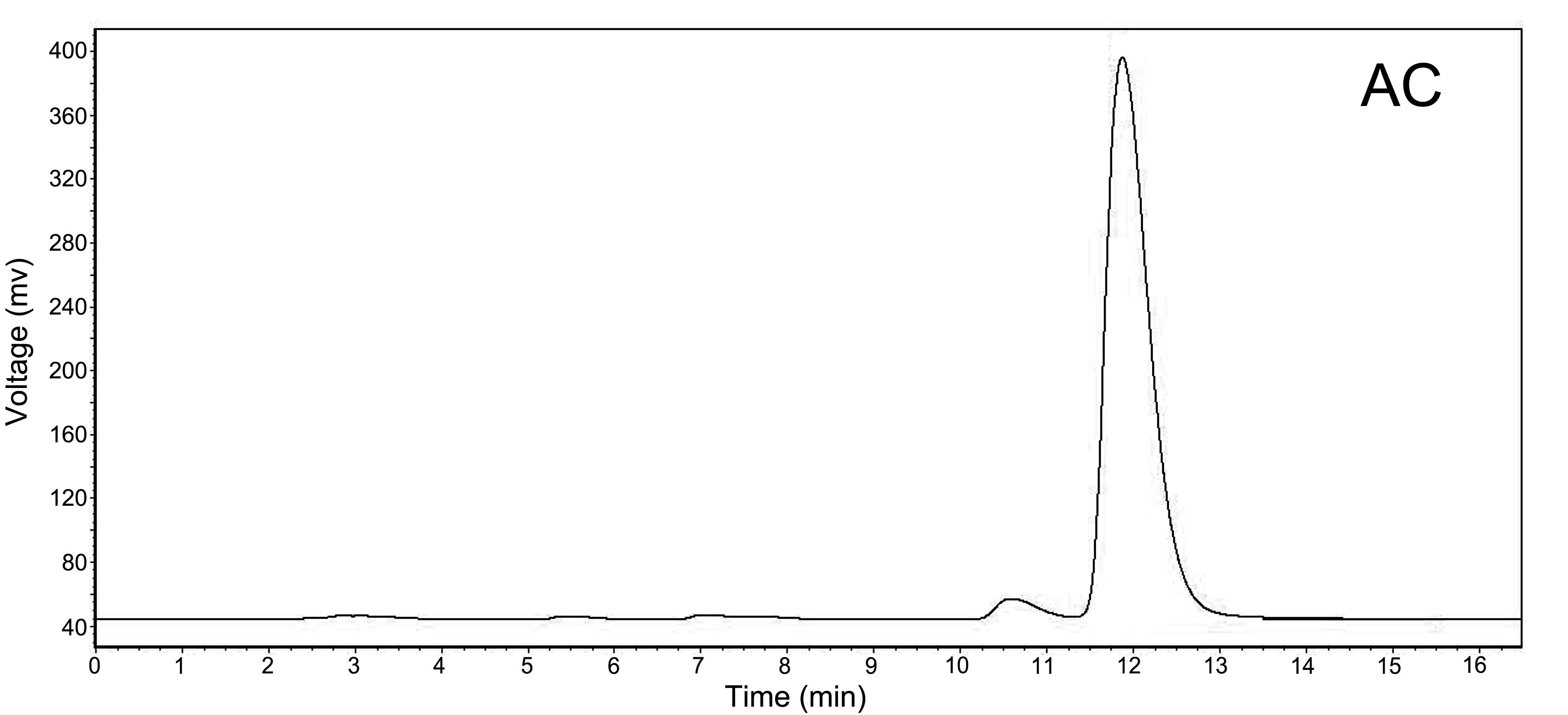

Supplement: Figure S6 — HPLC chromatogram of AC. (HPLC) [file pone.0095909.s006.hplc]

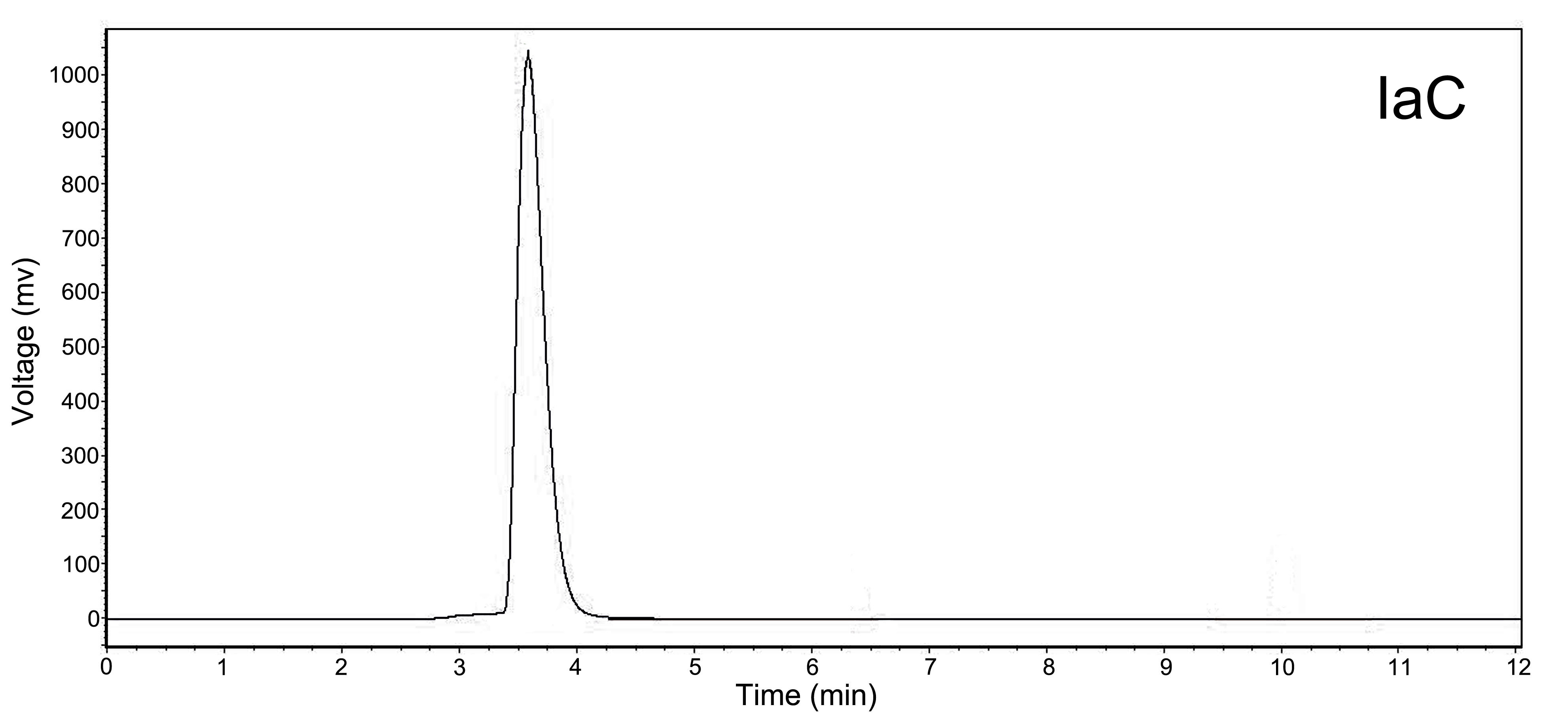

Supplement: Figure S7 — HPLC chromatogram of IaC. (HPLC) [file pone.0095909.s007.hplc]

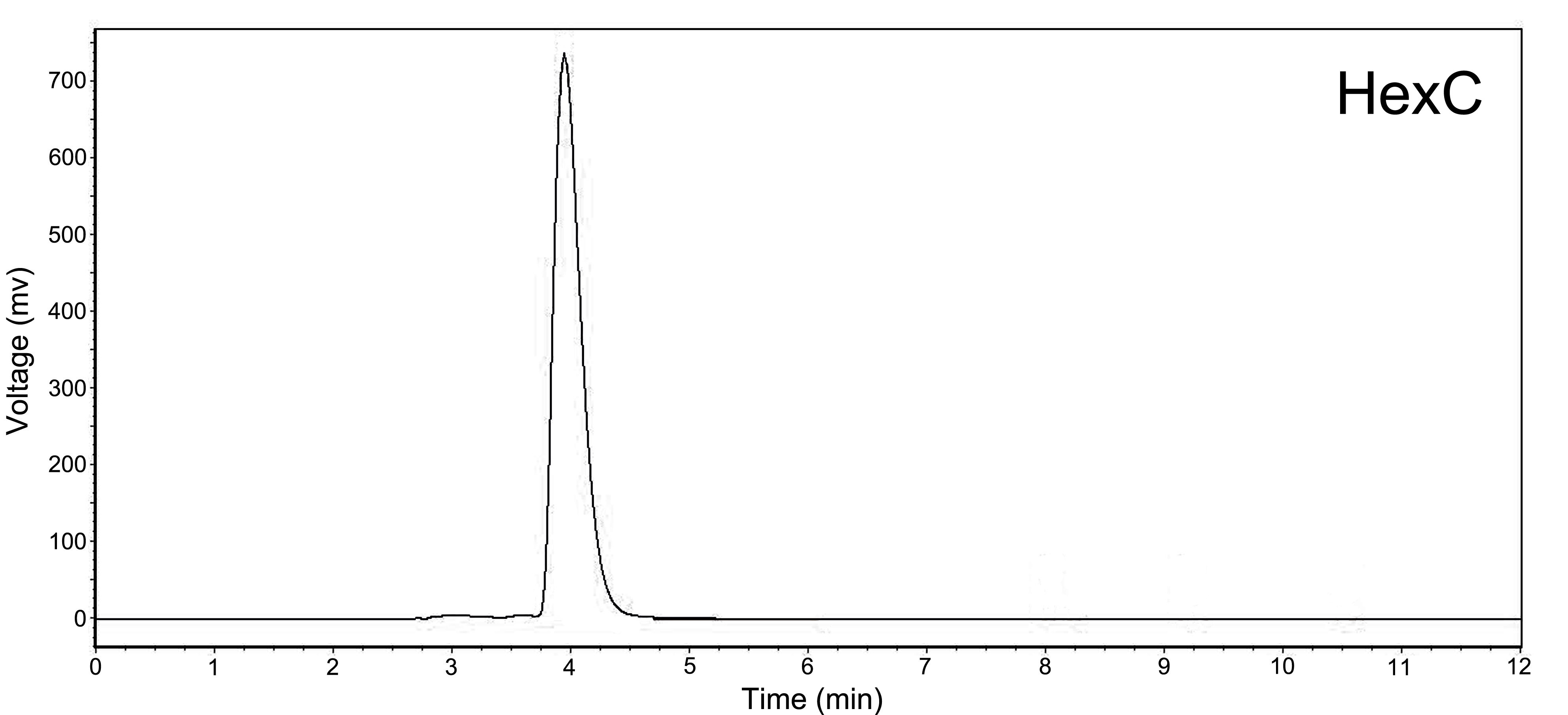

Supplement: Figure S8 — HPLC chromatogram of HexC. (HPLC) [file pone.0095909.s008.hplc]

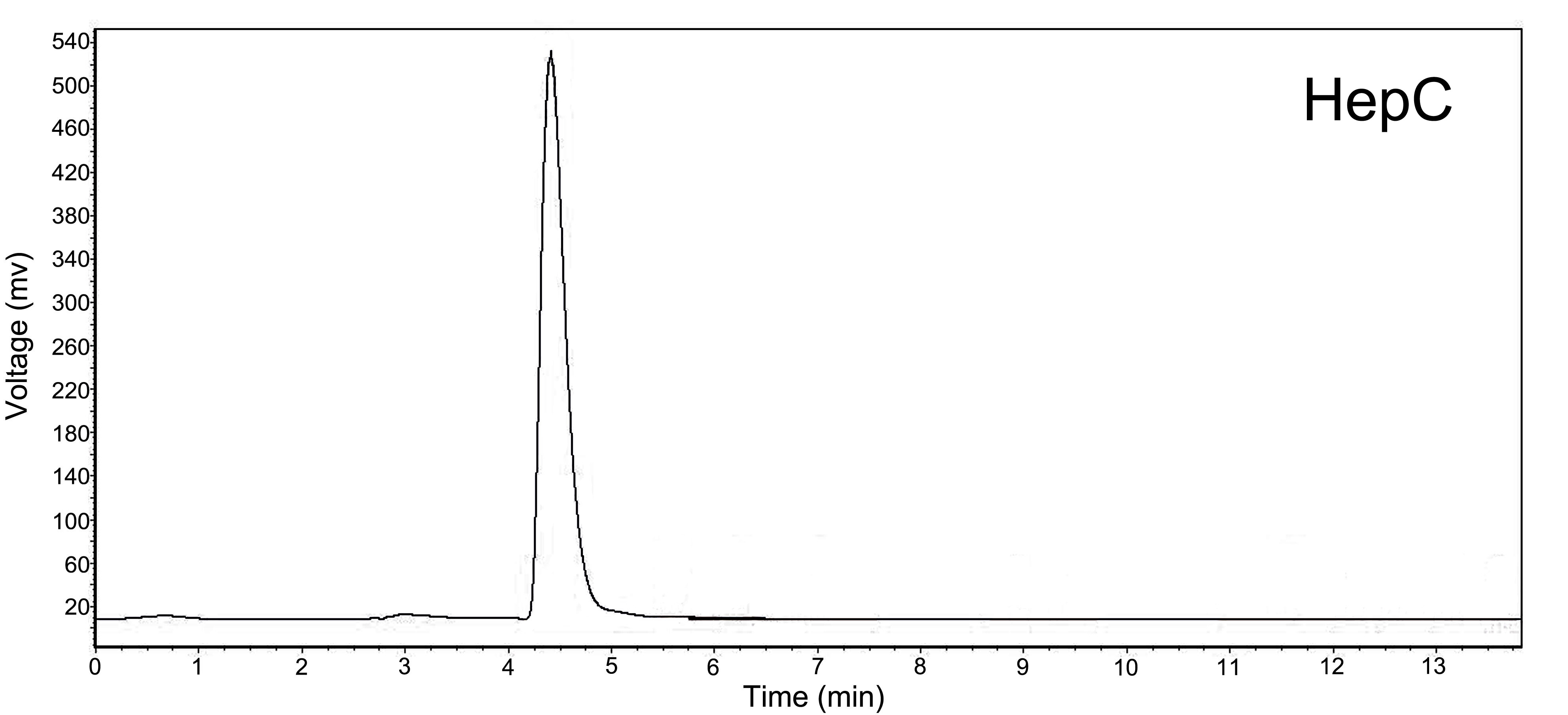

Supplement: Figure S9 — HPLC chromatogram of HepC. (HPLC) [file pone.0095909.s009.hplc]

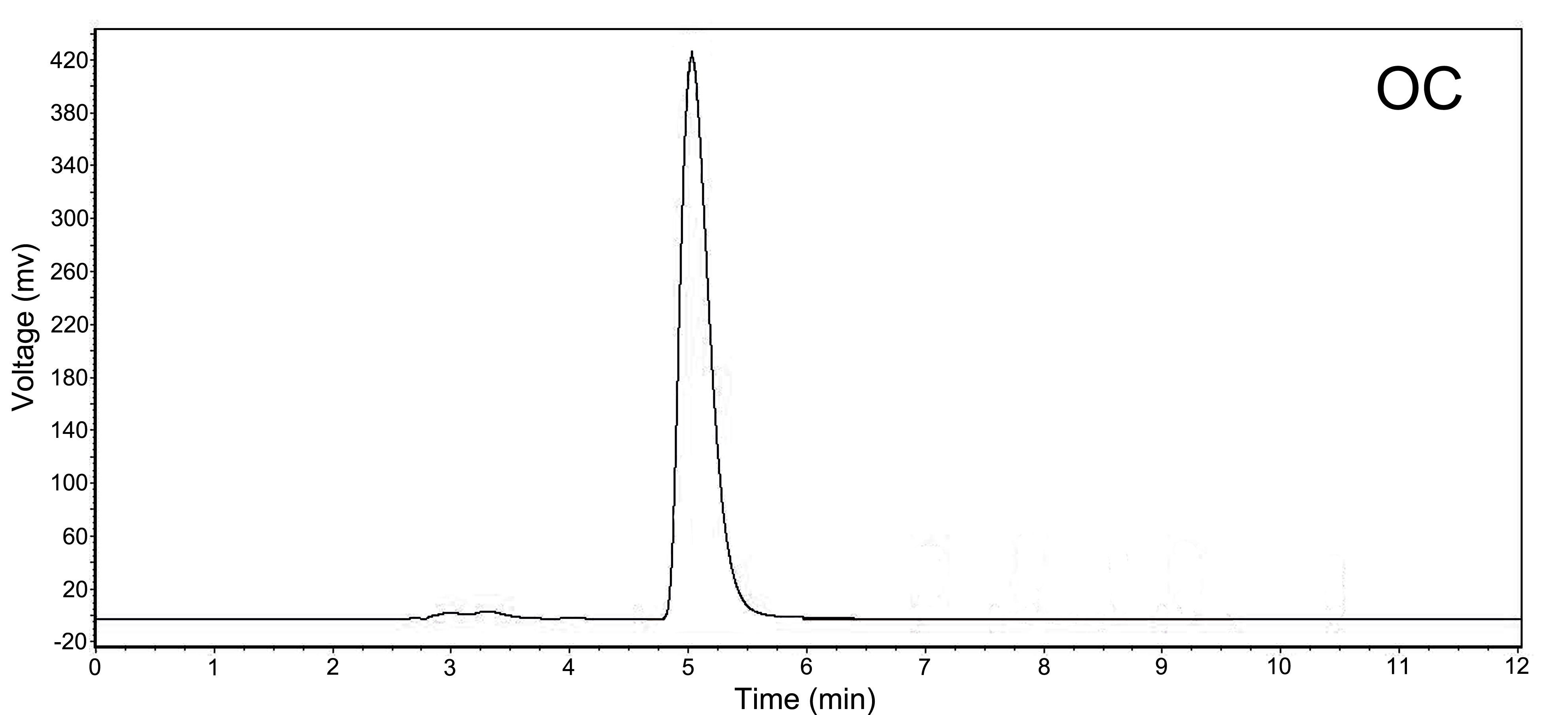

Supplement: Figure S10 — HPLC chromatogram of OC. (HPLC) [file pone.0095909.s010.hplc]
